# Is It Safe to Perform Revision Hip Arthroplasty Without Suction Drains?

**DOI:** 10.7759/cureus.39682

**Published:** 2023-05-30

**Authors:** Hafiz Muhammad Umer, Hafiz Javaid Iqbal, Nisarg Shah, Harshil Vora, Aatif Mahmood, Tim Board

**Affiliations:** 1 Trauma and Orthopedics, Stepping Hill Hospital, Manchester, GBR; 2 Trauma and Orthopedics, Countess of Chester Hospital, Chester, GBR; 3 Trauma and Orthopedics, Wrightington Hospital, Wigan, GBR

**Keywords:** drain, revision hip arthroplasty, haematoma, transfusion rate, prosthetic joint infection, blood loss, suction drains, revision hip replacement

## Abstract

Background

There is a number of studies showing no significant benefit of using drains after primary hip arthroplasty. However, there is no consensus in the literature about the usage of drains in revision hip replacement. This study aims to assess the effect of drains in revision hip arthroplasty.

Methods

A retrospective analysis was performed of all consecutive revision hip replacement surgeries performed at our unit over a five-month period (November 2018 to March 2019). Case notes, laboratory investigations, and operative records were reviewed. The effects of drains on postoperative hemoglobin (Hb), transfusion rate, and complications were analyzed.

Results

Overall, 92 patients were analyzed who underwent revision hip replacement during the study period. There were 46 male and 46 female patients with a mean age of 72 years. Aseptic loosening was the most common indication for the revision (41 patients) followed by instability (21 patients), infection (11 patients), and periprosthetic fractures (eight patients). Seventy-two patients had no drains while suction drains were used in 20 patients. Both groups were similar regarding age, sex, and indications for revision surgery. There was a significantly higher drop in postoperative Hb in patients with drains than those without drains (33 g/L: 27 g/L, p=0.03). There were significantly more blood transfusions in patients with drains as compared to those without drains (15%: 8%, relative risk 1.8, and odds ratio 1.94). There was no difference in both groups with regard to returning to the theater.

Discussion

Use of suctions drains in revision hip surgery was associated with increased postoperative blood loss and increased requirement for postoperative blood transfusion. Revision hip surgery without routine usage of suction drains did not increase wound complications.

Conclusion

Revision surgery without routine usage of drains is safe and may decrease postoperative blood loss and the rate of transfusion.

## Introduction

The use of closed suction drains (CSD) following total joint replacement has been an established practice in Orthopedic Surgery as advocated by Waugh and Stinchfield [1]. The rationale behind using CSD is to reduce wound complications such as hematomas, deep tissue infection, and post-operative pain and improve wound healing [2].

Randomized control trials [2,3] have not shown any benefits of routinely using CSD in primary hip replacement surgery. On the contrary, Overgaard et al. [4] concluded that CSD may lead to greater blood loss by eliminating the tamponade effect of hematoma and may allow retrograde bacterial contamination. Another recent meta-analysis showed that CSD failed to provide any benefit with regard to infection, functional recovery, and other complications [5] in primary arthroplasty.

In contrast to primary surgery, revision can vary from simple liner exchange to extensive dissection, extended trochanteric osteotomy, and full implant exchange. Surgical timing can be prolonged with more blood loss and more potential wound complications [6-8]. Advocates of CSD would cite this level of dissection and exposure as a need for the use of drains.

However, current literature shows insufficient evidence regarding the routine use of CSD in revision total hip arthroplasty (THA) [9]. A previous randomized controlled trial (RCT) conducted by Fichman et al. showed no benefit of CSD in revision THA [10]. However, this trial was limited, with small numbers. The use of CSD is still routine in many hospitals after revision hip replacement. In our institute, some surgeons routinely use CSD after revision hip replacement while others have discontinued using drains. This study aimed to compare the safety of revision hip replacement surgery with or without the routine use of CSD.

## Materials and methods

We retrospectively reviewed all consecutive patients undergoing revision THA at our institute (a tertiary care center) between November 2018 and March 2019. The surgeries were performed by fellowship-trained surgeons experienced in revision arthroplasties. Data were collected on consecutive patients using case notes, operative records, laboratory investigations, and clinic letters. Data regarding patient demographics, reasons for revision surgery, postoperative blood loss, need for transfusion, and post-operative complications were collected.

Revision arthroplasty procedures were performed using either standard trochanteric osteotomy or the posterior approach depending upon the surgeon’s choice. All patients were given antibiotics at induction and tranexamic acid as per hospital protocols. Two suction drains were used according to the surgeon’s preference. The drains were then removed 24-48 hours after the surgery. Postoperatively, patients were transfused if hemoglobin (Hb) was less than 80g/L.

Statistical analysis was performed using SPSS 22.0 software (SPSS Inc., Chicago, IL). For the continuous normally distributed variables, the Student's t-test was used to compare the mean of the two groups. Mann-Whitney U test was used for non-parametric continuous data. The Chi-Square test was used for categorical data. The odds ratio and relative risks were calculated. For all comparisons, a p-value of less than 0.05 was selected to represent significance.

This study did not require additional patient contact and as such this project was performed as a service evaluation. The project was approved by the institutional audit and clinical governance department.

## Results

A total of 100 patients underwent revision hip arthroplasty during the study period. However, eight were excluded due to insufficient data availability. Analysis was performed on the remaining 92 patients. The mean age of the patients was 72 years (range: 38-92 years) with an equal male-to-female ratio. Over two-thirds were admitted as elective patients, and the rest were either emergency admissions or urgent transfers from another trust (Table 1).

**Table 1:** Basic demographics

	Number of patients
Total No of patients	100
Excluded (incomplete data)	8
Data analyzed	92
Male:female	46:46
Elective admissions	74
Non-elective admissions	18

Just fewer than 50% were revised due to aseptic loosening. Other indications were instability, Prosthetic joint infections (PJI), and periprosthetic fractures (PPFs). The patients were divided into two groups, i.e., CSD group and the “No-Drain used” group. There was no difference in basic patient demographics, male-to-female ratio, and indications for surgery in both groups (Table 2).

**Table 2:** Basic comparison of the two groups *PPF: Periprosthetic infection

	No Drains used	Drains used (CSD)	P-value
N (Total no.)	72	20	
Male:Female	36:36	10:10	1.0
Age	72(38-92)	73(50-86)	0.6
Elective	56(86.7%)	18(90%)	0.3
Infection	9 (12.5%)	2(10%)	1.0
Instability	15 (21%)	4(20%)	1.0
PPF*	6(8.3%)	1(5%)	1.0
Aseptic loosening	33(46%)	12(60%)	0.3

Blood loss was calculated as the difference between preoperative and postoperative hemoglobin (Hb - g/L) levels. The patients in CSD group had significantly more blood loss (mean Hb drop - 33g/L) as compared to the “No-Drain used” group (mean Hb drop - 27g/L) (P =0.039). There was a non-significant trend toward a lower complication rate (return to theatre and postoperative infection) in the no-drain group (p=0.84) (Table 3).

**Table 3:** Post-operative outcomes comparison

	No Drains used	Drains Used (CSD)	P-value
Preoperative Hb (g/L)	129.2(99-169)	128(94-148)	1.0
Post-operative Hb(g/L)	102(64-139)	94(73-120)	0.03
Mean Drop in Hb (g/L)	20	33	
Wound Infections	3(4%)	1(5%)	0.84
Return to theatre	3(4%)	1(5%)	0.84

Similarly, patients with drains had a high requirement for postoperative blood transfusion. Fifteen percent of patients in the CSD group required transfusion whereas only 8% of those in the “No-Drain used” group required transfusion (odds ratio of 1.94 and relative risk of 1.8).

## Discussion

Although the trend of using drains in primary hip and knee arthroplasties is on the decline, drains are still commonly used in revision hip arthroplasties [10]. Our study showed no benefit of using suction drains in revision hip surgery. Based on our results, usage of suction drains was associated with a significant drop in post-operative Hb and increased requirement for postoperative transfusions. The rate of complications in terms of return to theatre and postoperative infection were lower in the non-drain group. However, this difference was not significant.

Several studies have discussed the relationship between blood loss, the need for postoperative transfusion, and its association with CSD in primary hip arthroplasty. Walmsley et al. [10] performed an RCT in 552 patients undergoing primary hip arthroplasty (577 hips) and concluded that the use of a drain did not influence the postoperative levels of Hb, the revision rates, Harris hip scores, the length of hospital stay or the incidence of thromboembolism. The rate of transfusion after operation in the drained group was significantly higher than those patients where the suction drains were not used (p < 0.042). A meta-analysis by Zhou et al. [5] in primary total hip arthroplasty showed reduced blood loss and reduced need for transfusion in the “No-Drain used” group. Similarly, a previous RCT by Finchman et al. [10] on revision total hip replacement showed significantly low postoperative Hb and significantly higher requirements of transfusion in patients with suction drains. Our results are similar to those of Finchman et al. [10]. In a small prospective comparative study of 40 patients, Matsuda et al. [11] demonstrated that drains did not increase the rate of anemia and blood transfusions after cementless primary total hip arthroplasty.

The use of a drainage system was previously also advocated for reducing hematoma formation and infection [1,12]. Hematomas may increase skin tension and decrease tissue perfusion providing an ideal space for bacterial growth. Alexander et al. [13] discussed that wound fluids provide an ideal medium for bacterial growth because they are deficient in opsin protein and thus reduce the chance of phagocytosis. Zeng et al. [14] used ultrasound to measure the thickness of the hematoma and found that the mean thickness was greater in the “No-Drain used” group. However, they did not find any difference in wound healing in both groups.

Although the infection rate in joint replacement has significantly decreased over the years from approximately 3%-4% to 0.3% [15] due to various measures, it remains a dreaded complication. Studies have suggested that drainage systems may provide access for retrograde migration of infection [16]. Gunterberg et al. [15] showed that cultures from drain tips and drain tracks were frequently positive. Raves et al. [17] showed that retrograde migration of bacteria may occur in 20% of patients with CSD by 72 hours. Despite these findings, Finchman et al. [10] did not find differences in infection rates in CSD and non-CSD groups. We noted in our study that one patient (5%) in the CSD group had a postoperative infection and three patients (4%) in the non-CSD group had an infection and thus failed to establish a linkage of infection with drains. However, this could be possibly due to the much smaller number of patients with CSD in our study.

The strengths of our study include analysis of consecutive patients, matched basic patient demographics and similar indications of revision surgery in both groups, the reasonable overall number of patients as compared to previous studies in revision hip surgery, and appropriate statistical measures showing the differences between the two groups. Our study has got some limitations. It is a retrospective comparative study, 8% of patients were excluded due to the non-availability of complete data, the number of patients was much smaller in the CSD group and estimated blood loss was only calculated using pre-operative and post-operative Hb values.

## Conclusions

Despite these limitations, in light of our findings and the existing evidence, usage of CSD failed to show significant benefit in revision hip replacement surgery. The usage of suction drains was associated with a significant drop in postoperative hemoglobin and increased requirement for transfusion. Further larger and multicentered studies are needed to establish the issues related to drain usage in revision hip surgery.
